# Correlation between muscle mass and handgrip strength in digestive cancer patients undergoing chemotherapy

**DOI:** 10.1002/cam4.2238

**Published:** 2019-05-21

**Authors:** Johanna Moreau, Marie‐Amélie Ordan, Coralie Barbe, Camille Mazza, Marine Perrier, Damien Botsen, Mathilde Brasseur, Christophe Portefaix, Yohann Renard, Barbara Tallière, Eric Bertin, Christine Hoeffel, Olivier Bouché

**Affiliations:** ^1^ Ambulatory Care Unit Reims University Hospital Reims France; ^2^ Department of Biostatistics Reims University Hospital Reims France; ^3^ Departement of Gastroenterology and Digestive Oncology Reims University Hospital Reims France; ^4^ Department of Radiology Reims University Hospital Reims France; ^5^ CReSTIC, Reims University Reims France; ^6^ Department of General and Digestive Surgey Reims University Hospital Reims France; ^7^ Artificial Nutrition Unit Reims University Hospital Reims France; ^8^ Department of Nutrition, Endocrinology, and Diabétology Reims University Hospital Reims France

**Keywords:** dynapenia, sarcopenia, muscle strength, muscle mass, digestive system neoplasms

## Abstract

**Background:**

FIGHTDIGO study has shown the feasibility of handgrip strength (HGS) measurements in 201 consecutive digestive cancer patients undergoing chemotherapy.

**Objective:**

This study focuses on a secondary aim of FIGHTDIGO study: the relationship between muscle mass and HGS.

**Design:**

Two consecutive bilateral measures of HGS were performed using a Jamar dynamometer before the start of each chemotherapy. The highest value was chosen for final evaluation. Dynapenia (loss of muscle strength) was defined as HGS < 30 kg (men) and < 20 kg (women). Muscle mass was measured at lumbar level (L3) on Computed Tomography (CT) scans performed less than 3 weeks before or after the measurement of HGS. Muscle mass loss was defined by skeletal muscle index (SMI) < 53 cm^2^/m^2^ (in men with a body mass index (BMI)> 25 kg/m^2^), < 43 cm^2^/m^2^ (in men with a BMI < 25 kg/m^2^), and < 41 cm^2^/m^2^ (in women regardless of BMI). Sarcopenia was defined by the association of a dynapenia and a loss of muscle mass.

**Results:**

A total of 150 patients were included in this analysis (mean age: 65.6 ± 10.9 years, 87 males (58%), colorectal cancer (47.3%), metastatic stage (76.7%)). A total of 348 CT scans were evaluated. For the 348 measurements, mean SMI and HGS were 41.8 ± 8.7 cm^2^/m^2^ and 32.1 ± 11.0 kg, respectively. Muscle mass loss, dynapenia, or sarcopenia were reported at least once, in 120 (80%), 45 (30%), and 30 (20%) patients, respectively. SMI was significantly correlated with HGS (Pearson coefficient = 0.53, *P* < 0.0001). At concordance analysis, 188 dyad SMI/HGS (54%) were in agreement (Kappa = 0.14 [95% CI, 0.07‐0.21]).

**Conclusion:**

Correlation between the measurements of HGS and SMI is strong but the concordance between dynapenia and muscle mass loss is poor. Further studies should be performed to confirm the diagnostic thresholds, and to study the chronology of dynapenia and loss of muscle mass.

## 1 INTRODUCTION

The association between denutrition and cancer has become a major concern worldwide during the last century.^1^ Digestive cancers are among the ten most diagnosed cancers.^2^ In this specific population, malnutrition is common, and is present in about 39% of colorectal cancers, 44% of esophagus and/or stomach cancers, and 67% of pancreatic cancers.^3^ As malnutritrion is responsible for a high morbidity and mortality rate, its screening and treatment are of paramount importance.^4^

Definition of malnutrition relies on a low body mass index (BMI), and/or on an unintentional weight loss, and/or on hypoalbuminemia without any inflammatory syndrome.^5^, ^6^ However, these three criteria do not totally reflect the complex physiopathology of malnutrition and its impact. For many years, more investigations have been carried out in the area of muscle mass loss, which occurs in 80% of patients with cancers, and is a first step towards malnutrition.^7^

Muscle mass alone cannot be interpreted without taking into account its function, which is represented by muscle strength. Sarcopenia is defined as the association of age‐related loss in skeletal muscle mass, as well as loss of muscle function (dynapenia or performance).^8^, ^9^

Handgrip strength (HGS) evaluation using a handgrip dynamometer has been widely studied. Strength physiologically declines with age.^10^, ^11^, ^12^ Weak strength, called dynapenia,^13^ predicts the risk of mortality from all causes when measured during mid‐life in general population.^10^ Moreover, dynapenia seems to be a factor of disability,^14^ nosocomial infection,^15^ and length of hospital stay 16 in elderly people. In oncological context, low HGS is associated with cancer‐related fatigue,^17^ poor quality of life,^18^ postoperative complications,^19^ chemotherapy toxicity, 20 and high mortality.^21^ The FIGHTDIGO study has shown the feasibility and acceptability of routine HGS measurements in digestive cancer patients undergoing ambulatory chemotherapy.^4^ The association between pretherapeutic dynapenia and chemotherapy‐induced dose‐limiting neurotoxicity has also been reported.^22^

Currently, the relationship between muscle mass and muscle strength remains poorly known in oncology. The second aim of the FIGHTDIGO study was to analyze the relationship between the HGS evaluated using Jamar dynamometer and the muscle mass evaluated on Computed‐Tomography (CT) scan.

## 2 MATERIALS AND METHODS

### 2.1 Study design and participants

The prospective monocentric FIGHTDIGO study was conducted in the ambulatory cancer unit (UMA‐CH) in Reims teaching hospital (CHU) in France.^4^ Study population included patients older than 18 years of age, having a primary digestive cancer regardless of its stage, and undergoing cytotoxic chemotherapy and/or biotherapy. Patients who could not give their consent, did not understand the HGS, had a history of neuro‐muscular disorder and/or had appointed a health care proxy, or whose CT scanner could not be analyzed were excluded. The patients were recruited from May 18, 2016 to November 18, 2016, and then were followed up for 6 months. They were asked to perform the HGS test on each of their appointment to the unit, before the start of their treatment (every week, every two weeks or more, depending on the chemotherapy regimen).

Patients performed CT scans during this period in routine care. In this study, only the CT scans performed less than three weeks before or after a HGS test were analyzed.

### 2.2 Ethical approval

Informed written consent was obtained for each enrolled patient in the trial. The FIGHTDIGO study was approved by the ethics committee (Committee for the Protection of Person EST I DIJON, March 25.2016) and was registered June 13, 2016 in Clinicaltrials.gov (NCT02797197).

### 2.3 Outcome

The aims were to evaluate the quantitative correlation between the measurements of HGS using Jamar dynamometer and muscle mass on the CT scans, and also the qualitative concordance between dynapenia, muscle mass loss, and sarcopenia according to consensual classification.^9^

### 2.4 HGS measurement

HGS was measured with a hydraulic Jamar dynamometer. Position 2 was used among the 5 possible handle‐positions. Every patient was seated comfortably in a chair. The shoulder of the upper limb holding the dynamometer was in an adduction position, the elbow flexed to 90 degrees, and the forearm and wrist in a neutral position. The other upper limb was placed alongside the body and relaxed. During the examination, patients were verbally encouraged as to try and obtain their best score. Four measurements were determined: each one had to last three seconds; patients had to perform the first two measurements in a row: one with the dominant hand and the other one with the non‐dominant hand; a one‐minute break was then respected before repeating the last two measurements. The highest value was chosen for final evaluation. According to the European Working Group on Sarcopenia, dynapenia was defined as HGS < 30kg (men) and < 20 kg (women).^8^

### 2.5 CT scans and muscle mass assessment

**Figure 1:**
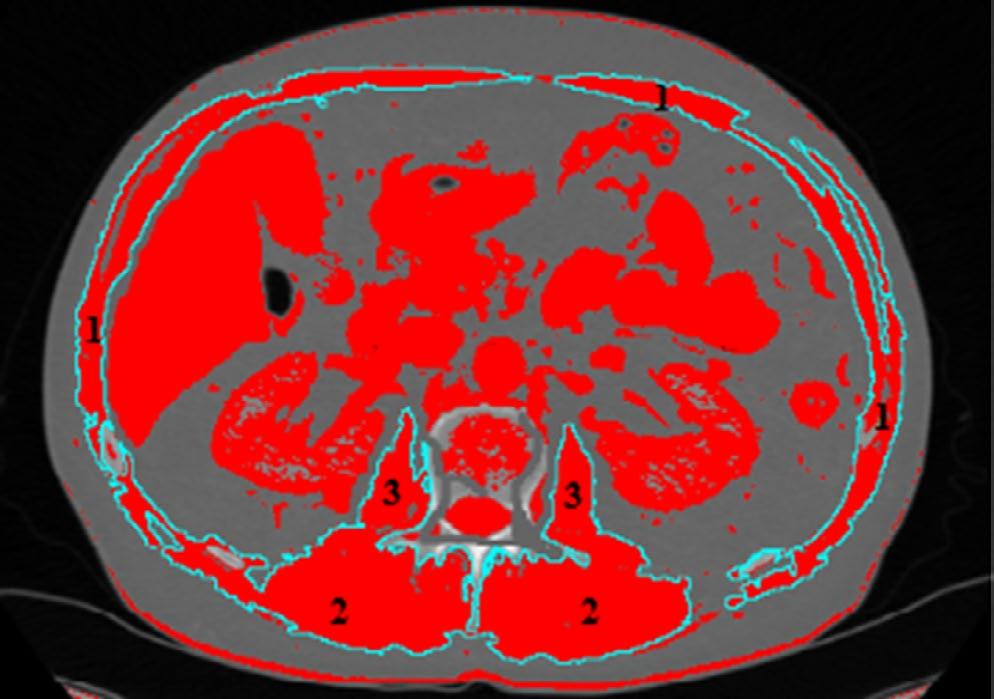
Selection of lumbar muscle areas with specific thresholds at third lumbar vertebra (L3). Regions of interest (inturquoise blue) corresponding to rectus abdominis, quadratus lumborum, transversus abdominis, external and internal oblique (1), paraspinal (2), and psoas muscles (3). Image from ImageJ software

Body mass index (BMI) was calculated [weight (kg)/height (m^2^)]. Skeletal muscle index (SMI) was defined as total muscle area (TMA) measured on an axial section of CT scan through the third lumbar vertebra (L3), at a level where both pedicles were visible using a preestablished density threshold (−29 to + 150 Hounsfield units) for skeletal muscles. SMI was then normalized to height and expressed in cm^2^/m^2^.^23^ The muscles in the L3 region include psoas, erector spinae, quadratus lumborum, transversus abdominis, external oblique, internal obliques, and rectus abdominis muscles.^24^, ^25^, ^26^, ^27^, ^28^, ^29^ After a period of training with a radiologist, a resident in gastroenterology who was unaware of the HGS values, measured TMA (cm^2^) using manual segmentation on a dedicated posttreatment station. ImageJ software v1.46r, a free public domain software developed by the National Institute of Health (NIH) was used (Figure 1).^30^, ^31^ According to the definition proposed by Martin et al, patients were considered as presenting a loss of muscle mass as follows: SMI (TMA at L3 divided by height squared) < 53 cm^2^/m^2^ in men with a BMI > 25 kg/m^2^, < 43 cm^2^/m^2^ in men with a BMI < 25 kg/m^2^, and < 41 cm^2^/m^2^ in women regardless of their BMI.^23^

### 2.6 Association between muscle mass and HGS

To perform this correlation, SMI measurements performed on CT examinations were associated with the HGS measurements using binomial analysis. The delay between the two measurements should be less than 3 weeks. Patients were considered to be sarcopenic in case of an association of both dynapenia and muscle mass loss.

### 2.7 Statistical analysis

Quantitative variables were expressed as mean ± standard deviation (SD) or as median +[range], and qualitative variables as numbers (percentages).

Patient's characteristics were compared between patients with and without available CT scans in an univariate analysis using Students’ *t* tests for continuous variables or using Fisher exact tests for qualitative variables.

Association between SMI and HGS was studied using Pearson's correlation coefficient. Concordance between dynapenia and loss of muscle mass was analyzed using Kappa (κ) coefficient with κ values of 0.00‐0.20 indicating "poor", 0.21‐0.40 indicating "fair", 0.41‐0.60 indicating "moderate", 0.61‐0.80 indicating "good", and 0.81‐1.00 indicating "excellent" agreement. A *P* value < 0.05 was considered statistically significant. All analyses were performed using SAS version 9.4 (SAS Inc, Cary, NC).^32^

## 3 RESULTS

### 3.1 Description of the population

**Figure 2:**
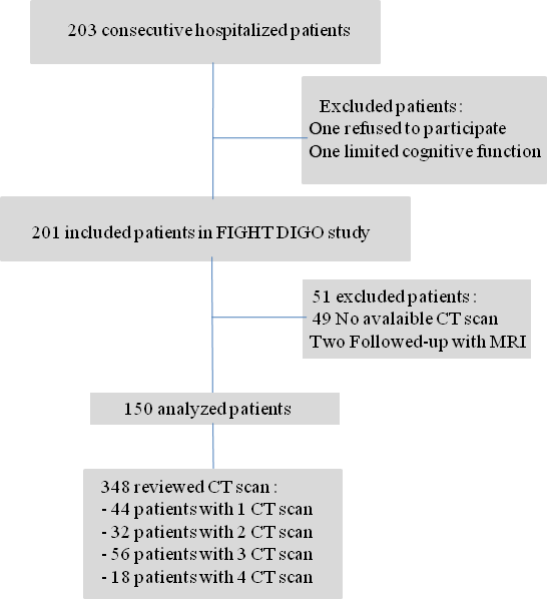
Patient flow chart: patient identification, inclusion, and exclusion steps

Among the 201 consecutive patients included in FIGHTDIGO study, CT scan examinations were unavailable in 51 patients. Finally, 348 CT scans were analyzed in 150 patients as shown in the patient flow chart (Figure 2). Table 1 shows baseline patient characteristics. Mean age was 65.6 (± 10.9) years. Colo‐rectal cancer was the most frequent cancer (n = 71, 47.3%). The majority of the patients was treated for metastatic tumor (n = 115, 76.7%).

**Table 1:** Baseline population characteristics

Characteristics	FIGHTDIGO Population (n=201)	Patients with at least one assessable CT scan (n=150)	No assessable CT scan (n=51)	Univariate Analysis *P* value
Age, mean ± SD	65.5 ± 10.8	65.6 ± 10.9	65.1 ± 10.8	0.80
Gender, N (%)				0.92
Men	117 (58.2)	87 (58.0)	30 (58.8)	
Women	82 (41.8)	63 (42.0)	21 (41.2)	
BMI, mean ±SD	25.0 ± 5.1	24.9 ± 4.9	25.3 ± 5.5	0.56
Leg edema, N (%)	4 (2.0)	4 (2.7)	0 (0.0)	0.57
Serum albumin, mean ±SD	38.6 ± 4.5	38.4 ± 4.6	39.4 ± 4.1	0.15
C‐reactive protein, mean ±SD	15.9 ± 32.8	17.8 ± 37.1	11.1 ± 15.5	0.09
Previous oncologic surgery, N (%)	128 (63.7)	87 (58.0)	41 (80.4)	0.004
mGPS, N (%)				0.29
0	99 (55.0)	68 (51.5)	31 (64.6)	
1	68 (37.8)	54 (40.9)	14 (29.2)	
2	13 (7.2)	10 (7.6)	2 (6.2)	
G8 score, mean ± SD	12.4 ± 2.1	12.1 ± 2.6	13.2 ± 2.2	0.12
Primary tumor, N (%)				0.35
Colon and rectum	103 (51.2)	71 (47.3)	32 (62.7)	
Oesphagus	8 (4.0)	5 (3.3)	3 (5.9)	
Stomach	22 (10.9)	16 (10.7)	6 (11.8)	
Cholangiocarcinoma	11 (5.5)	9 (6.0)	2 (3.9)	
Pancreas	44 (21.9)	38 (25.3)	6 (11.8)	
Small intestine	3 (1.5)	2 (1.3)	1 (2.0)	
Neuroendocrine tumor	8 (4.0)	7 (4.7)	1 (2.0)	
Adenocarcinoma, unknown primitive tumor	2 (1.0)	2 (1.3)	0 (0.0)	
Stage, N (%)				<0.0001
Local	40 (19.9)	19 (12.7)	19 (41.2)	
Locally advanced	23 (11.4)	16 (10.7)	7 (13.7)	
Metastatic	138 (68.7)	115 (76.7)	23 (45.1)	
Type of treatment, N (%)				0.004
Chemotherapy alone	147 (73.1)	101 (67.3)	46 (90.2)	
Biotherapy alone	4 (2.0)	4 (2.7)	0 (0.0)	
Chemotherapy and biotherapy	50 (24.9)	45 (30.0)	5 (9.8)	
Indication, N (%)				<0.0001
Adjuvant	47 (23.4)	23 (15.3)	24 (47.1)	
Neoadjuvant	10 (5.0)	7 (4.7)	3 (5.9)	
Palliative	144 (71.6)	120 (80.0)	24 (47.1)	
First line, N (%)	132 (65.2)	92 (61.3)	40 (78.4)	0.03

Abbreviations: BMI, body mass index; mGPS, modified Glasgow Prognostic Score; N, number; SD, standard deviation.

Comparison between baseline characteristics of patients with at least one available CT scan (n = 150) and of patients without available CT scan (n = 51) are showed in Table 1. Patients with at least one available CT scan had significantly more metastatic tumors (76.7% versus 45.1%; *P* < 0.0001), had had previous surgery more often (58.0% versus 80.4%; *P* = 0.004), were more often treated with biotherapy (32.7% versus 9.8%; *P* = 0.001), were more often undergoing palliative treatment (80.0% vs 47.1%; *P* < 0.0001), and were less often undergoing first line treatment (61.3% vs 78.4%; *P* = 0.03).

### 3.2 Relationship between HGS and muscle mass.

A total of 348 dyad HGS/CT scan were analyzed. The number of CT scans per patient is shown in Figure 2. Forty‐four patients had only one CT scan and only 18 patients had four CT scans. The median time between the measurements of HGS and SMI was 8 days [0 ; 21].

SMI and HGS quantitative and qualitative results are reported in Table 2. For the 348 measurements, mean SMI and HGS were respectively 41.8 (± 8.7) cm^2^/m^2^ and 32.1 (± 11.0) kg. Among the 150 studied patients, muscle mass loss, dynapenia, or sarcopenia were reported at least once, in 120 (80%), 45 (30%), and 30 (20%) patients, respectively.

**Table 2:** Skeletal muscle index (SMI) and handgrip muscle strength (HGS) results (348 measurements in 150 patients)

Dyad CT scan/HGS	
SMI, mean± SD	41.8 ± 8.7
SMI loss, N (%)	58 (16.7)
HGS, mean± SD	32.1 ± 11.0
HGS loss (dynapenia), N (%)	192 (55.2)
Sarcopenia (HGS and muscle mass loss), N (%)	50 (14.4)

Abbreviations: HGS, handgrip strength; SMI, skeletal muscle index.

**Figure 3:**
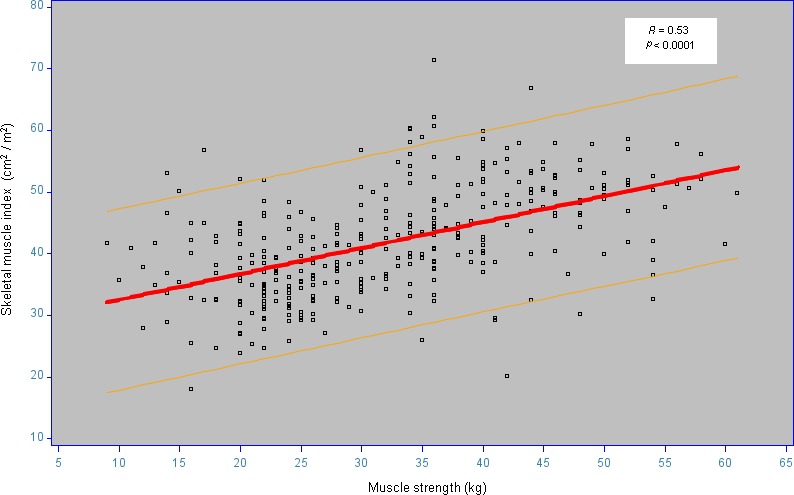
Correlation between handgrip muscle strength (HGS) and skeletal muscle index (SMI). The regression curve is in red. The yellow curves surround 95% of values. The letter r is Pearson coefficient

SMI was correlated with HGS (*r* = 0.53, *P* < 0.0001) (Figure 3). At concordance analysis, 188 dyad SMI/HGS (54%) were in agreement (κ = 0.14 [95% CI), 0.07‐0.21]). Dynapenia and loss of muscle mass were present in 45 dyads (12.9% of the 348 dyads). Dynapenia and loss of muscle mass were absent in 143 dyads (41.1% of the 348 dyads). Of the 348 analyzed CT scan, eight patients had edema without any significant influence on SMI (38.7 ± 8.3 cm^2^/m^2^ for patients with edema versus 41.9 ± 8.7 cm^2^/m^2^ for patients without edema, respectively ( *P* = 0.24).

## 4 DISCUSSION

To our knowledge, this is the first study to evaluate and show a good correlation between the measurement of HGS and the muscle mass, both pivotal parameters of sarcopenia definition,^8^, ^9^ in cancer patients undergoing a cytotoxic chemotherapy. SMI was indeed positively correlated with HGS (r = 0.53, *P* < 0.0001). In contrast, the concordance was poor between dynapenia and muscle mass loss according to consensual classification. Muscle mass loss was more frequently present than dynapenia.

Another study evaluating the relationship between HGS and muscle mass suggested that high levels of HGS may not be closely related to greater muscle mass,^33^ nor low levels of HGS related to muscle mass loss. However, HGS was positively associated with the dependent variable (muscle mass).^33^ The population in that study was different from ours as it involved women survivors of breast cancer in Columbia). Moreover, another technique, namely tetrapolar bioelectrical impedance analysis was used to determine muscle mass 33 as well as the fatty mass (% and kg), the muscle mass (% and kg), and the total mass (kg). Furthermore, no patient had clinically detectable edema, a condition that could have affected resistance and reactance.

The weak agreement between SMI and HGS confirms that the issue of sarcopenia and dynapenia cutoffs and measurement methods, which vary across literature, have not yet been unanimously established.^8^, ^12^, ^23^, ^24^, ^29^, ^34^, ^35^, ^36^, ^37^, ^38^

Measurement of SMI is only quantitative and does not take into account the quality of the muscle. Edema could overrate SMI measurements. We did not evidence any impact of edema on the SMI though, but the number of patients with edema was very low in our series (8 out of 150). Skeletal muscle density (SMD) reflects muscular quality. Dolan et al showed that low SMI and myosteatosis (low SMD) were significantly associated with survival in colorectal patients undergoing surgery.^39^ Yet, they did not study muscle strength although it is noteworthy that sarcopenia is defined by a loss of strength and muscle mass.^8^, ^9^

Since SMI obtained with CT scan is the gold standard evaluating sarcopenia, but is burdened by its costs and ionizing radiation exposure.^40^, ^41^, ^42^ Alternate methods for measurement of muscle mass may be used including as dual energy X‐ray or tetrapolar bioelectrical impedance analysis.^33^, ^43^

Several studies have examined the relationship between evaluation of the different components of the diagnosis of sarcopenia diagnosis,^33^, ^44^, ^45^, ^46^, ^47^, ^48^, ^49^, ^50^ yet the relationship and chronology between muscle strength and muscle mass is still poorly known. The analysis of this chronology was not planned to be included in the FIGHTDIGO study because CT examinations were not performed in a systematic way. The time interval between the CT scans were indeed very variable and only 18 patients had 4 CT scans (maximum number of performed CT examination). Moreover, the patients were included at different time points during their follow‐up and were likely to have received several cycles and lines of chemotherapy before the measurements. Muscle mass loss appeared to be far more frequent (80%) than dynapenia (30%) and sarcopenia (20%). Loss of skeletal muscle mass could therefore precede that of muscle strength.

Limitations of this study were the heterogeneity of the population, the sub‐group analysis, and the small number of chemo‐naïve patients undergoing CT follow‐up.

In conclusion, correlation between measurements of HGS and SMI was strong but the concordance between dynapenia and muscle mass loss according to consensual classification was poor. It was very interesting how an easily measurable clinical tool like HGS correlated well with SMI. Muscle mass loss could precede dynapenia. Further studies should be performed so as to confirm diagnostic threshold values of the different modalities and to study the chronology of dynapenia and of muscle mass loss.
